# A predictive score to identify hospitalized patients' risk of discharge to a post-acute care facility

**DOI:** 10.1186/1472-6963-8-154

**Published:** 2008-07-22

**Authors:** Martine Louis Simonet, Michel P Kossovsky, Pierre Chopard, Philippe Sigaud, Thomas V Perneger, Jean-Michel Gaspoz

**Affiliations:** 1 Service of General Internal Medicine, University Hospitals, Geneva, Switzerland; 2 Groupe de Recherche et d'Analyse en Systèmes et Soins Hospitaliers, Department of Medicine, University Hospitals, Geneva, Switzerland; 3 Division of Primary Care Medicine, University Hospitals, Geneva, Switzerland; 4 Quality of Care Service, University Hospitals, Geneva, Switzerland

## Abstract

**Background:**

Early identification of patients who need post-acute care (PAC) may improve discharge planning. The purposes of the study were to develop and validate a score predicting discharge to a post-acute care (PAC) facility and to determine its best assessment time.

**Methods:**

We conducted a prospective study including 349 (derivation cohort) and 161 (validation cohort) consecutive patients in a general internal medicine service of a teaching hospital. We developed logistic regression models predicting discharge to a PAC facility, based on patient variables measured on admission (day 1) and on day 3. The value of each model was assessed by its area under the receiver operating characteristics curve (AUC). A simple numerical score was derived from the best model, and was validated in a separate cohort.

**Results:**

Prediction of discharge to a PAC facility was as accurate on day 1 (AUC: 0.81) as on day 3 (AUC: 0.82). The day-3 model was more parsimonious, with 5 variables: patient's partner inability to provide home help (4 pts); inability to self-manage drug regimen (4 pts); number of active medical problems on admission (1 pt per problem); dependency in bathing (4 pts) and in transfers from bed to chair (4 pts) on day 3. A score ≥ 8 points predicted discharge to a PAC facility with a sensitivity of 87% and a specificity of 63%, and was significantly associated with inappropriate hospital days due to discharge delays. Internal and external validations confirmed these results.

**Conclusion:**

A simple score computed on the 3rd hospital day predicted discharge to a PAC facility with good accuracy. A score > 8 points should prompt early discharge planning.

## Background

Efficient discharge planning is an important component of hospital care. The goals of discharge planning are to ensure continuity in health care beyond hospital discharge and optimal use of hospital beds [1].

Current evidence suggests that discharge delays are an important cause of inefficient hospital use. In general internal medicine wards, 30% of all hospital discharges may be delayed for non-medical reasons, and these delays represent 17% of all hospital days [2]. At our Department of internal medicine, we observed similar results: 28% of all hospital days were rated as inappropriate and half of these were due to delays related with the discharge process [3]. The most frequent cause for discharge delays was waiting for a bed in a post-acute care (PAC) facility. Thus, early identification of patients who will require such post-acute care could reduce inappropriate hospital use by planning their discharge earlier.

Several studies have tried to develop screening instruments of problems linked with the discharge process in patients hospitalized for acute medical conditions [4-8]. However, these instruments vary markedly in their outcome measures (hospital length of stay (LOS), inappropriate hospital days, need for post-discharge services, hospital readmissions) and do not specifically address the issue of delays due to discharge to a PAC facility.

The purposes of this study were to determine, among patients hospitalized for an acute medical condition, which factors predicted their risk of being discharged to a PAC facility, and to identify which assessment time of the corresponding variables had the best predictive power and was the most efficient. We compared two strategies of early detection: the first used data collected within the first 24 hours of admission (day 1) and the second data collected on the 3rd hospital day (day 3). Then, we developed and validated a simple score based on our preferred model that stratified patients' risks of discharge to a PAC facility for use as a screening instrument in ordinary clinical settings.

## Methods

This prospective study was divided into two parts: the development phase (derivation and internal validation of two predictive models, followed by construction of a score) from January 1st to April 30th 2001; and the prospective validation phase (prospective validation of the score on a new cohort of patients) from October 1st to November 30^th ^2003.

### Setting

The Geneva University Hospitals is an 1100-bed urban teaching hospital with 224 medical beds, serving a large community as well as a referral population. Only medical patients with an acute condition are admitted in these medical beds. At our study site, discharge planning services are provided in accordance with the medical plan of care. Patient's physician and primary nurse manage routine or uncomplicated discharges. For complicated or non-routine discharges, we use a consultative discharge planning model in which physicians, ward nurses and social workers work together to assess, coordinate and implement the patients' discharge plan. In addition, a weekly formal multidisciplinary ward round is held for the assessment of all patients' discharge plan.

### Patients

Patients were recruited on admission to the general internal medicine wards. To be included in the derivation and validation cohorts, patients had to be discharged to home or to a PAC facility (skilled nursing facility or inpatient rehabilitation facility). Comatose and terminally ill patients on admission were excluded. We also excluded from further analysis patients who died in hospital after enrolment, were transferred to other acute care hospital settings, or were discharged to the nursing home in which they lived prior hospital admission. The study was part of a hospital quality improvement protocol and was approved by the institutional ethics committee

### Outcomes

Our main outcome variable was the patient's discharge destination, i.e. whether the patient was discharge to home or was discharge to a PAC facility. In this study indeed, as the most frequent cause for discharge delays in our service was waiting for a bed in a PAC facility, we were concerned with discriminating patients unable to return home and eventually transferred to a PAC facility from those returning home with (or without) formal (or informal) help. Therefore, we did not consider referral to home care as a PAC destination.

Secondary outcomes, which were not used in modeling, included: LOS, number of inappropriate hospital days, and number of inappropriate hospital days due to discharge delays, as determined by the Appropriateness Evaluation Protocol (AEP) and Delay Tool [2,9]. The AEP uses 27 criteria to assess the appropriateness of each hospital day (11 relate to medical services/procedures, seven to nursing/life support services, and nine to clinical characteristics of the patient necessitating close observation). Once a day has been identified as medically unnecessary (i.e. no information in the medical record corresponding to any of the 27 explicit criteria), the Delay Tool allows the description of factors potentially responsible for such medically unnecessary episodes of care using a complementary list of reasons. The agreement of the research nurses on appropriateness of hospital days and on causes of delays was verified on a sample of 6 patients who totaled 52 hospital days (kappa = 0.90 for both appropriateness of hospital days and causes of delay).

### Baseline predictor variables

Two trained clinical nurse researchers carried out structured questionnaires with the patients and their family if necessary, the residents in charge of their care, and their primary nurses within 24 hours of admission and on the 3^rd ^hospital day. They collected demographic, clinical, and administrative data, as well as discharge diagnoses and treatment regimens from medical charts and hospital administrative databases. The baseline patient questionnaire included information about living situation, formal or informal home help, drug regimen prior to hospitalization, the number of hospitalizations or medical visits during the prior 3 months, self-reported basic activities of daily living (ADLs) and self-reported instrumental activities of daily living (IADLs) two weeks prior to admission. The seven basic ADLs (including feeding, grooming, dressing, toileting, bathing or taking a shower, walking and transferring) and the five IADLs (including travelling via car or public transportation, food or clothes shopping (regardless of transport), meal preparation, housework, medication use (preparing and taking correct dose)) were dichotomously rated as dependent or independent by research nurses. Dependency was defined as partial or total need of assistance from another person. On admission, the following data were also collected: type of admission (from home vs. internal hospital transfer), number of active medical problems (defined as a medical condition that required active either diagnostic or therapeutic strategies, or monitoring), Charlson comorbidity index [10], Mini-Mental State Examination (MMSE) score for patients aged 65 or more [11], sensory deficits, and presence of decubitus ulcers. Orientation and behavioral disturbances were also recorded by asking the nursing staff if patients were adequate in their time and space orientation, if they could recognize their relatives, and if they were agitated, confused, wandering or aggressive. On the 3rd hospital day, the nursing staff reassessed and rated the seven basic ADLs, as well as orientation or behavioral disturbances, and the presence of decubitus ulcers.

### Statistical analysis

#### Power calculation

Information extracted from internal databases let us expect a transfer rate of 30% to a PAC facility. The sample size needed to estimate this proportion with a 95 percent confidence interval of 0.05 is 323. We therefore aimed at this sample size, increasing our recruitment in order to take into account ineligible patients and dropouts.

#### Derivation of the two predictive models, construction of a score and internal validation

Univariable analyses, using chi-square tests or student t-tests, were performed to assess the association between demographic and clinical characteristics and discharge to a PAC facility. To assess independent associations with the outcome of interest, all variables were included into 2 multivariable logistic regression models, separately for the two data collection times (day 1 vs. day 3). Two-way interaction terms were tested as well. Backward elimination, in which the least significant variable was discarded at each step, was then used, until all remaining variables in the models reached a significance level of 0.05 or less. The accuracy of both multivariable models to identify patients discharged to a PAC facility was assessed by means of their ROC curves.

A simple integer score was computed from the best final parsimonious multivariable logistic regression model, assigning points in proportion to the regression coefficients. Then, a cross-validation procedure was performed in order to assess the degree of over-fitting of the prediction model to the development sample [12]. The sample was divided at random into 10 parts; a prediction model was developed on 9 tenths and applied to the remaining tenth; this was repeated 10 times, rotating the validation set. The ability of cross-validated score to predict discharge to a PAC facility was examined by comparing the areas under their receiver operating curves (AUC) with that obtained from the naive prediction score. We finally defined post-hoc score categories on the basis of increasing risk of discharge to PAC facility.

#### Validation of the score

The predictive score created in the derivation cohort was calculated in the prospective validation cohort on the 3^rd ^hospital day by the research nurses who kept the ward staff blinded to its results. The score capacity to predict discharge to a PAC facility was compared between the prospective validation cohort and the initial derivation cohort by means of AUCs.

Finally, the association between score's risk categories and LOS, as well as the number of inappropriate hospital days and the number of inappropriate hospital days due to awaiting for post discharge facilities were assessed in both cohorts by means of Kruskall-Wallis equality of populations rank test. Analyses were performed using Stata release 8 (Stata Corporation, College Station, Texas).

## Results

### Derivation cohort

During the derivation enrolment phase, we recruited 412 patients, of whom 63 (15.3%) were excluded from further analyses: the main reasons were death during hospital stay (20, 4.9%); transfer to other acute care hospital settings, such as intensive care unit or surgical wards (24, 5.8%); and patients' discharge to the nursing home in which they lived prior to hospital admission (19, 4.6%). Excluded patients were older than enrolled patients (mean age, 75 vs. 65 years; p < 0.001), were more likely to live alone, and reported a higher number of disabilities in both ADL and IADL tasks (5.8 vs. 2.5 disabilities; p < 0.001).

Of the 349 patients evaluated on admission and enrolled in the study to build the day 1 model, 245 (70%) were discharged home and 104 (30%) to a PAC facility. Patients discharged to a PAC facility were older and were more likely to live alone than patients discharged home (Table 1); if not living alone, they were less likely to receive home help from their partners, had a higher number of formal home care providers and reported a higher number of functional impairment (ADLs and IADLs) prior to admission. In addition, such patients were more frequently admitted through internal hospital transfer, had a higher number of drugs prescribed before admission; they also had a lower Mini Mental State Examination score and had a higher number of active medical problems on admission. No significant differences were observed in the proportions of patients discharged home versus to a PAC facility for principal diagnoses such as cardio-vascular, pulmonary, rheumatic or neurological diseases, while patients with oncology disease were less likely to be discharged to a PAC facility (17%; p < 0.005).

**Table 1:** Patients' characteristics of the derivation cohort (n = 349)

Characteristics of the patients			p
	Discharged to a PAC facility	Discharged home	
	n = 104 30%	n = 245 70%	
Age: mean (± SD)	71 (14)	62 (18)	< 0.001
Number of men (%)	47 (47%)	113 (52%)	0.45
Number of patients living alone (%)	62 (60%)	101 (41%)	0.002
Number of patients whose partner provided home help (%)	27 (26%)	109 (44%)	0.001
Number of formal care providers at home: mean (± SD)*	2.0 (1.1)	1.6 (1.0)	0.04
Number of informal care providers at home: mean (± SD)^†^	1.4 (0.6)	1.3 (0.5)	0.45
Number of drugs prior admission: mean (± SD)	5.0 (3.3)	4.1 (3.2)	0.02
Number of hospital stays in the past 3 months: mean (± SD)	0.4 (0.7)	0.5 (1.1)	0.60
Number of emergency room visits in the past 3 months: mean (± SD)	0.2 (0.7)	0.2 (0.6)	0.79
Number of patients admitted from other hospital services (%)	29 (28%)	28 (11%)	<0.001
Number of self-reported ADL^‡ ^disabilities upon admission: mean (± SD)	1.8 (2.2)	0.5 (1.2)	<0.001
Number of self-reported IAD^§ ^disabilities upon admission: mean (± SD)	2.5 (2.0)	1.1 (1.6)	<0.001
Number of active medical problems on admission: mean (± SD)	3.6 (1.5)	2.7 (1.3)	<0.001
Charlson comorbidity index: mean (± SD)	2.1 (2.0)	1.8 (2.0)	0.20
Mini Mental State score on admission: mean (± SD)^||^	24.5 (4.3)	25.7 (3.5)	0.05
Number of ADL disabilities on day 3: mean (± SD)**	3.8 (2.8)	1.2 (2.2)	<0.001
Length of hospital stay: mean (± SD)	13.2 (7.8)	10.1 (6.4)	< 0.001

*only for people receiving professional home care (n = 104)

^†^only for people receiving informal home care (n = 271)

^‡^ADL: Activities of daily living

^§ ^IADL: Instrumental activities of daily living

^||^only for people aged 65 and over (n = 175)

**only for people assessed on day 3 by nurse staff (n = 299)

Since 30% (n = 104) of the 349 enrolled patients received professional home care before their admission, we compared these patients with those not receiving such help (n = 245). Not surprisingly, these patients were older (74 vs. 61 yrs; p < 0.001), had more disability in ADLs (1.4 vs. 0.7; p < 0.001), IADLs (2.4 vs. 1.2; p < 0.001), and comorbidities assessed by the Charlson comorbidity index (2.4 vs. 1.7; p = 0.008); they were also more likely to live alone (64% vs. 39%; p < 0.001) and less likely to be helped by a partner (25% vs. 45%; p < 0.001). In terms of sex, and active medical problems, both groups were similar. Among these 104 patients, 64 (62%) were discharged home and 40 (38%) to a PAC facility. Both groups were however similar except in terms of disability in IADLs (2.0 vs 3.1; p = 0.005).

After admission, 50 patients left our service within 3 days: 41 (11.7%) were discharged home and 9 (2.6%) to a PAC facility. These 50 patients were significantly younger than patients who remained 3 days or more (57 years ± 18 vs. 66 ± 16; p < 0.001). The remaining 299 patients (85.7%) were evaluated on the 3rd hospital day and enrolled to built the day 3 model; 95 of them (31.8%) were later discharged to a PAC facility.

### Comparison of the two predictive models and construction of the score

Logistic regression modeling with backward elimination yielded slightly different models according to the time of data collection (day 1 vs. day 3) (Table 2). In both models, the number of active medical problems on admission and the inability of the patient's partner to provide home help were significant predictors of discharge to a PAC facility. Older age, an increasing number of functional impairment (ADLs + IADLs; 12 items) and being admitted through internal hospital transfer were significant predictors on day 1, but no longer on day 3. When age groups (18–34 years, n = 20 (6.7%); 35–49 years, n = 32 (10.7%); 50–64 years, n = 65 (20.7%); 65–79 years, n = 121 (40.5%) and 80–93 years, n = 61 (20.4%)) were used in the day 3 model instead of continuous scale, we also found no significant association, (p = 0.47). On day 3, dependencies for only three activities among the 12 ADL and IADL tasks were significant predictors (medication self-management before admission, bathing and transferring from a bed to a chair at day 3). Areas under the ROC curves reached 0.81 (95% CI: 0.76 – 0.86) for day 1 model and 0.82 (95% CI: 0.76 – 0.87) for day 3 model. We compared the ability of the 2 models to correctly allocate patients. The proportion of patients correctly allocated reached 78.5% for the Day-1 model and 77.9% for the Day-3 model. We also performed the Hosmer-Lemeshow goodness of fit test with 8 degrees of freedom on both day 1 and day 3 models. P values were respectively 0.37 and 0.21, indicating good fit for each model.

**Table 2:** Multivariate logistic regression modeling of variables associated, on days 1 and 3, with discharge to a PAC facility.

Variables	Adjusted odds-ratio (95%CI)			
	Day 1	P	Day 3	p
	n = 349		n = 299	
Number of medically active problems on admission (per additional problem)	1.4 (1.1 – 1.7)	0.003	1.3 (1.0 – 1.6)	0.02
Inability of patients' partner to provide home help	3.4 (1.9 – 6.3)	<0.001	2.5 (1.3 – 4.9)	0.006
Number of disabilities; ADL* + IADL^† ^(per additional disability out of 12)^‡^	1.3 (1.1 – 1.4)	<0.001	-	-
Age (per additional decade)	1.3 (1.1 – 1.5)	0.004	-	-
Type of admission (hospital internal transfer vs. from home)	2.0 (1.0 – 4.1)	0.05	-	-
Dependency for bathing/taking a shower^§^	-	-	2.8 (1.3 – 6.0)	0.007
Dependency for transfers bed/chair^§^	-	-	2.8 (1.3 – 6.2)	0.008
Inability in medication self-management before admission^‡^	-	-	2.5 (1.2 – 5.2)	0.01

* ADL: Activities of daily living; ^† ^IADL: Instrumental activities of daily living;

^‡^patients self-report upon admission; ^§ ^nurse staff assessment on day 3

Since 50 out of 349 patients (14%) left the hospital before day 3, we considered that assessing the likelihood of being discharged to a PAC facility on day 1 was not an efficient strategy. Therefore, construction of the predictive score was based on the model developed from data collected in 299 patients on day 3. Table 3 illustrates how points were attributed to each component of the score. We attributed points in proportion to the values of the unexponentiated logistic regression coefficients of the predictive variables, because we wanted to express the true quantitative ratio between the weights of the variables in the model. Of note that confidence intervals not overlapping zero in unexponentiated coefficients are equivalent to confidence intervals not overlapping one if odds-ratios had been used. For the variable number of medical active problem, a continuous one, the regression coefficient was 0.24. Since all other regression coefficients had a value around 1, we decided to give them a value 4 times the value of the coefficient associated with active medical problems. Therefore, 1 point was attributed for every additional active medical problem and 4 points for every other variable. When variables in the prediction model were allowed to vary independently, AUC reached 0.82; when variables were added to form the score, AUC was also 0.82. The scores ranged from 0 to 24. After cross-validation, AUC was 0.81, ruling out substantial over-fitting of the score.

**Table 3:** Logistic regression coefficients and corresponding points attributed to day 3 variables significantly associated with discharge to a PAC facility.*

Variable	Logistic regression coefficient	95%CI	p	Point score
Active medical problems (per additional problem)	0.24	0.04 – 0.45	0.02	+1
Inability of patients' partner to provide home help	0.93	0.26 – 1.59	0.006	+4
Dependency for bathing	1.04	0.29 – 1.79	0.007	+4
Dependency for transfers (bed/chair)	1.05	0.28 – 1.83	0.008	+4
Inability in medication self-management before admission	0.92	0.19 – 1.64	0.01	+4

* The coefficient corresponds to the natural logarithm of the odds-ratio

At a cut-point of 8 or more, the score correctly classified 71% of the patients discharged to a PAC facility and predicted the risk of being discharged to a PAC facility with a sensitivity of 87%, a specificity of 63%, a positive predictive value of 53% and a negative predictive value of 91%. At a cut-point of 16 or more, the score correctly classified only 42% of the patients discharged to a PAC facility and predicted this outcome with a sensitivity of 42%, a specificity of 95%, a positive predictive value of 80% and a negative predictive value of 78%. Therefore, the score was chosen as the cut-point of 8 and more in an attempt to maximize both sensitivity and specificity. Indeed, our goal was to early identify patients at risk of not returning home in order to take appropriate actions towards these patients. Consequently, we were more willing to accept a high number of false positives (i.e patients identified as candidates for transfer to a PAC facility who subsequently would not experience it) than a high number of false negatives, (i.e. patients not identified as candidates for transfer to a PAC facility who subsequently would require it).

We finally analyzed misclassified patients using our algorithm. Out of the 158 patients with a score of 8 points or more, 75 subsequently return home. These patients were similar to patients discharged to PAC facility (n = 83) in terms of living situation, formal or informal home help, comorbidities and active medical problems. However, these patients were younger (68 years vs, 74 years; p = 0.014) and had less disabilities in ADLs (1.1 vs.1.9; p = 0.012) and IADLs (1.9 vs. 2.8;p = 0.006). False negatives patients were less frequent (n = 12) and did not significantly differ from patients returning home with the same score (less than 8 points).

### Validation of the score

**Figure 1:**
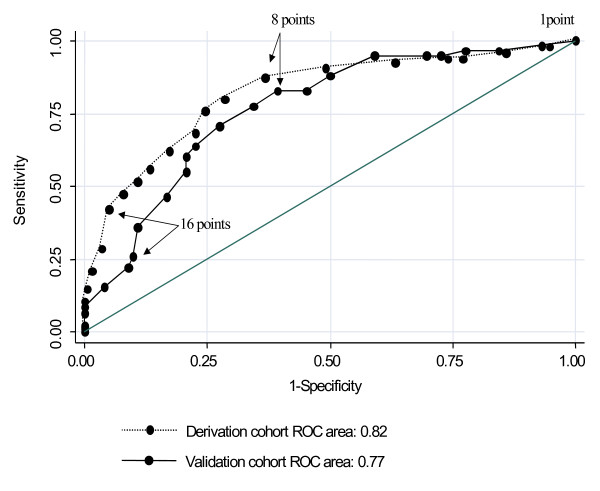
Predictive accuracy of the score.

Of the 183 patients recruited during the prospective validation phase, 22 (12%) were excluded from further analysis: 4 patients died during the hospital stay, 8 were transferred to other acute care hospital settings and 10 were discharged to the nursing home where they lived. Of the 161 remaining patients, 58 (36%) patients were discharged to a PAC facility and 103 (64%) home. Clinical characteristics of the 161 patients were similar to those of the derivation cohort except in terms of mean number of informal care providers at home (0.76 ± 0.6 for the validation cohort vs.1.04 ± 0.7 for the derivation cohort; p < 0.001), of mean Charlson comorbidity index (2.6 ± 2.4 vs. 1.9 ± 2.0; p < 0.001), and of mean number of impairment in the ADL's on the 3^rd ^hospital day (3.4 ± 2.4 vs. 2.3 ± 2.1; p < 0.001).). Their 3-day scores ranged from 1 to 22. The score's capacity to predict discharge to a PAC facility was higher in the derivation cohort (AUC = 0.82) than in the prospective validation cohort (AUC = 0.77) but the difference was not statistically significant (p = 0.31) (Figure 1).

Since death or transfer to other acute care hospital settings are not predictable on day 3, we checked whether including the 22 patients who experienced such outcomes in the validation cohort significantly modified our results. Mean 3-day score among them reached 11.2 ± 5.8 points vs. 10.7 ± 6.0 for patients discharged home or transferred to a PAC facility (p = 0.68). However, the score's capacity to predict discharge to a PAC facility was not significantly different in the validation cohort when these patients were included in the analysis (AUC = 0.74 vs. 0.77; p = 0.69). Therefore, for strict comparability purposes between the derivation and the validation cohorts, we decided to keep these 22 patients out of further analyses.

### Score categories and association with LOS and inappropriate hospital days

We arbitrarily stratified patients of the derivation cohort into three score categories according to the proportion of patients discharged to a PAC facility: 0 to 7 points (less than 10% of patients); 8 to 15 points (40%); and 16 points and over (80%). LOS, number of inappropriate hospital days, and number of inappropriate hospital days due to discharge delays significantly increased as the score categories increased (Table 4). We found similar associations in the validation cohort.

**Table 4:** Association between score categories and hospital use.

Score	Patients discharged to a PAC facility	LOS in days	Inappropriate hospital days	Inappropriate hospital days due to discharge delays
	n (%)	(mean ± SD)	(mean ± SD)	(mean ± SD)
a. Derivation cohort (n = 299)				
Less than 8 points n = 141 (47%)	12 (9%)	11.4 (± 6.6)	2.8(± 3.5)	0.2 (± 1.1)
8 to 15 points n = 108 (36%)	43 (40%)	12.6 (± 6.2)	3.6 (± 4.1)	1.5 (± 2.9)
16 points and more n = 50 (17%)	40 (80%)	14.5 (± 7.0)	4.8 (± 4.8)	3.6 (± 3.6)
P	<0.001*	0.005^†^	0.03^†^	<0.001^†^
				
b. Prospective validation cohort (n = 161)				
Less than 8 points n = 58 (36%)	7 (12%)	8.6 (± 5.9)	3.0 (± 3.7)	0.7 (± 2.3)
8 to 15 points n = 59 (37%)	24 (41%)	12.6 (± 7.2)	5.0 (± 5.5)	2.6 (± 4.6)
16 points and more n = 44 (27%)	27 (61%)	14.1 (± 9.7)	5.3 (± 6.5)	3.5 (± 5.6)
P	< 0.001*	<0.001^†^	0.08^†^	<0.001^†^

* Fisher's exact test; ^† ^Kruskal-Wallis test

## Discussion

We developed an accurate, objective and simple clinical prediction rule that identified, on the third hospitalization day, patients' risks of discharge to a post-acute care facility and that relied on readily available clinical parameters. The discriminative ability of the score was good, even when applied to an independent cohort of patients. Application of this tool may help clinical teams identify patients at high and low risk of transfer to a PAC. This in turn would allow better anticipation of patients' needs, and a smoother transition between acute care and post-acute care.

Discharge delays that unnecessarily prolong hospitalizations are often due to difficulties either in finding a bed in a PAC facility, or in organizing post-discharge health or social services, and to failure to initiate planning until discharge is imminent [2,3,13-18]. In addition, previous studies have demonstrated that older age, as well as cognitive and functional impairments, predict discharge delays [15,19,20] and increase hospital LOS [2,21-23]. The current study expands these findings in developing a screening tool for the early identification of medical inpatients who will not be able to directly return home after a hospitalization and who will be discharged to a PAC facility. Inability of a patient's partner to provide home help, inability to self-manage drug regimen, number of active medical problems on admission, dependency in bathing and in transfers from bed to chair on the 3^rd ^hospital day, were independent predictors of discharge to a PAC facility. A score built with these factors predicted such an outcome with reasonable accuracy and was significantly associated with increasing LOS, inappropriate hospital days, as well as inappropriate hospital days due to discharge planning delays.

Discharge planning screening method should have at least two objectives: first, to identify early and accurately patients who need help which requires specific discharge-planning procedures; and second, to minimize the number of inappropriate referrals for comprehensive discharge planning. With this rationale, many health care providers say that discharge planning should begin on the day of admission. Our findings corroborate previous research indicating that older age, social environment, and functional status assessed on admission are key factors to predict discharge destination after an acute hospital stay [21,22,24-27]. However, we found that screening on the third hospital day patients likely to be discharged to a PAC facility was as accurate but more effective than screening them within 24 hours of admission. Using readily available data and based on only five independent factors, the day-3 model was both more simple and easier to establish, and avoided the unnecessary screening of patients who left hospital within 48 hours. In addition, assessing functional autonomy on the 3^rd ^hospital day takes better into account the substantial worsening in functional status documented in many elderly patients in the days following hospital admission [28-31]. Finally, we observed that older age was no longer predictive of discharge to PAC facility in the day-3 model. This could be explained by the fact that patients who left hospital within 48 hours were notably younger than patients who were staying longer and therefore included in the day-3 model.

Screening tools for discharge delays of patients hospitalized for acute medical conditions have been developed previously [4,5,7,15]. However, our tool addressed one precise issue, the identification of patients who will be discharged to a PAC facility, and therefore at risk of discharge delays, while other tools predicted less focused or combined outcomes, such as non-medical hospital days [7,15], post-discharge problems [5], or the use of post-discharge medical services including formal home care [4]. More recently, two screening tools have been successfully developed to predict more focused outcomes, such as the risk of nursing home placement for older people [26], or the use of specialized hospital discharge planning services for medical and surgical patients [32]. However, differences in study populations and predicted outcomes make strict comparison between these studies and our screening tool difficult. Finally, with a sensitivity of 87% and a specificity of 63%, our tool reaches a reasonable predictive accuracy, while other published tools were similarly specific but often less sensitive and most of the time required complicated data collection falling outside routine care [5,7,26].

The association between high scores and inappropriate hospital use gives clues for future interventions. Since residents' ability to predict patients' site of living after hospital discharge is low [18,21], routine calculation of the score on the 3^rd ^hospital day by residents and ward nurses, as well as its systematic integration into the patient's medical charts, may help reduce inappropriate hospital use. Scores ≥ 8 points could alert the house-staff about difficulties in patients returning home, while components of such a high score may help them identify functional impairments that could benefit from the early intervention of physical therapists to prevent further functional decline or to restore function. They may also prompt assistance from social service to expedite organization of appropriate home help. If such measures are not possible or fail, high scores could trigger the house-staff to anticipate and organize earlier the timing of discharge to a PAC facility.

Misclassification of those patients using our algorithm could be viewed as a major weakness of our screening tool. Since we mostly wanted to be conservative and to avoid false negatives, i.e. patients in need of transfer to a PAC but not being identified as such, sensitivity was our main concern, and it was 87%. This led to a negative predictive value of 91%. Conversely, in our derivation cohort, 37% of patients with a score of 8 points or more were not subsequently discharged to a PAC facility. However, we strongly believed that early identification of those patients at risk of not returning home can act as an alarm, and engage specialized discharge planning services in a more timely and appropriate manner, potentially reducing the risk of subsequent transfer to a PAC facility. Applying such a discharge planning strategy could reduce patients' inappropriate hospital use without unduly increasing patient's transfer to a PAC facility.

Our study has several limitations. First, although similar to most European institutions, mean LOS at our hospital was long (11.3 days) compared with North American hospitals [17,33]. In addition, other institutions or other countries may face shorter discharge delays because of greater non-acute bed availability. However, despite a marked decrease in hospital LOS in the USA in the recent years, the proportion of inappropriate hospital days has changed only little and still culminates on the last days of care, because of patients waiting for post-discharge facilities or for post-discharge services to be organized [2,13,16]. Second, our derivation cohort was remarkably intact in terms of cognition, since only 20% of our patients were aged 80 years or more. This probably explains why impaired cognition was not associated with the risk of being discharged to a PAC facility in our score. Third, our score was measured by research nurses specifically appointed for the project: use of the same score in daily routine nursing activities may not yield the same predictive power. Fourth, our score did not identify patients who needed a transfer to a PAC facility but rather those likely to experience it, irrespective of its appropriateness. This precision is important since needs and ability to meet them may vary from one setting to the other. We also excluded patients transferred to the nursing homes were they lived because patients living in such institutions represent a group with different characteristics from patients living at home, notably in terms of living conditions and help provided. This could significantly modify and lower their need of post-acute care facility. We also excluded patients who died during hospital stay or who were transferred to other acute care settings, since death or transfer to other acute care hospital settings are not predictable on day 3. However, when we included these patients in our analysis, our results were not modified. Finally, we derived and validated our score at a single institution and only among medical patients admitted for an acute condition in general internal medicine wards. Although diagnoses such as stroke, congestive heart failure and pulmonary diseases are known to be associated with transfer to a PAC facility and turned out to be the most common ones in our sample, they did not increase prediction of transfer to a PAC facility. Because of these limitations, use of the score to other settings will require local validation.

## Conclusion

Our study provides a validated, simple, and easy-to-use prediction tool that predicts medical patients' risks of discharge to a PAC facility at the end of an acute hospital stay. Specific interventions targeted towards high-risk patients could save inappropriate hospital use.

## Competing interests

The authors declare that they have no competing interests.

## Authors' contributions

MLS conceived and designed the study, managed the protocol, supervised the acquisition of data, analysed and interpreted the data and wrote the manuscript. MPK conceived and designed the study, managed the protocol, supervised the acquisition of data, analysed and interpreted the data and wrote the manuscript. PC contributed to the protocol design, participated in data analysis and in the writing of the paper. PS collected the data and checked their accuracy. TVP contributed to the protocol design, gave expertise in statistical analysis, participated in data analysis and in the writing of the paper. J–MG contributed to the protocol design, analysed and interpreted the data and participated in the writing of the paper.

## Pre-publication history

The pre-publication history for this paper can be accessed here:


